# Delayed diagnosis of axial spondyloarthritis: the crucial role of primary care — how you can make a difference

**DOI:** 10.3399/bjgp25X740997

**Published:** 2025-02-28

**Authors:** Thomas A Ingram, Joe Eddison, Karl Gaffney, Raj Sengupta, Daniel Murphy, Toby Wallace, Sampada Bhide, Stephanie Cliffe, Lucy McCann, Jill Hamilton, Clare Clark, Dale Webb, on behalf of the Act on Axial SpA campaign

**Affiliations:** CEO, National Axial Spondyloarthritis Society, London.; CEO, National Axial Spondyloarthritis Society, London.; Rheumatology, Norfolk & Norwich University Hospitals NHS Foundation Trust, Norwich.; Consultant Rheumatologist and Clinical Lead for Axial Spondyloarthritis, Rheumatology, Royal National Hospital for Rheumatic Diseases, Bath.; Honiton Surgery, Honiton; Rheumatology, Royal Devon and Exeter NHS Foundation Trust, Exeter.; Derwent Practice, Malton.; Advanced Practice Physiotherapist and AP-FCP, Physiotherapy, Kingston Hospital NHS Foundation Trust, London.; Musculoskeletal Advanced Practice Physiotherapist, Physiotherapy, Midlands Partnership University NHS Foundation Trust, Stoke-on-Trent.; Advanced Physiotherapy Practitioner/First Contact Practitioner, Physiotherapy, South Warwickshire University NHS Foundation Trust, Warwick.; CEO, National Axial Spondyloarthritis Society, London.; CEO, National Axial Spondyloarthritis Society, London.; CEO, National Axial Spondyloarthritis Society, London.

## Introduction

Spondyloarthritis (SpA) is a term encompassing a family of chronic inflammatory diseases that share common clinical features such as axial skeletal involvement, peripheral musculoskeletal (MSK) manifestations (arthritis, enthesitis, and dactylitis), extra-musculoskeletal manifestations (EMMs: inflammatory bowel disease, psoriasis, and acute anterior uveitis), and an association with the HLA-B27 antigen.^1^^,^^2^ This group of diseases can be categorised into axial SpA (predominantly pain and inflammation of the axial skeleton) or peripheral SpA (predominantly pain and inflammation of the peripheral joints or entheses).^1^^,^^2^ Axial SpA is typified by involvement of the spine and sacroiliac joints and comprises radiographic axial SpA (formerly known as ankylosing spondylitis) and non-radiographic axial SpA.^1^^,^^2^ A hallmark characteristic of radiographic axial SpA is sacroiliitis visible on plain radiographs, whereas, in non-radiographic axial SpA, structural damage may not be present initially, but inflammation of the sacroiliac joints can be observed via magnetic resonance imaging.^1^^,^^2^

The principal symptom of axial SpA is chronic low back pain (frequently with inflammatory features);^2^ however, fatigue is also common.^3^ These symptoms often leave patients with functional impairment, sleep disturbance, work disability, mental health issues, and poor health-related quality of life.^3^ The estimated prevalence of axial SpA in the UK adult primary care population ranges from 0.3% to 1.2%^4^ and the majority (92%) of patients have an age at onset of <45 years (median 26 years [interquartile range 20–34]).^5^

Delayed diagnosis remains a worldwide problem in axial SpA.^6^^,^^7^ The reasons are multifaceted;^6^^,^^7^ however, in the UK a large proportion of diagnostic delay occurs once patients have entered the healthcare system.^8^ Here, we briefly describe the literature pertaining to diagnostic delay, the challenges and experiences in primary care, and the resources available to support primary care practitioners (PCPs) in the identification and referral of suspected axial SpA.

## Diagnostic delay

People living with axial SpA experience prolonged waits to receive a diagnosis, which denies them the opportunity to lead normal lives and access advanced therapies. A systematic review and meta-analysis estimated the mean delay to diagnosis to be 8.65 years in the UK,^6^ while Hay *et al* identified two studies that reported median delays of 5 and 6 years.^7^

Descriptive results from a systematic review of 21 studies suggested that delayed diagnosis of axial SpA was associated with significant clinical, economic, and humanistic burden.^9^ Specifically, individuals with a delayed diagnosis had worse disease activity and function, higher direct and indirect healthcare costs, and a greater likelihood of work disability and psychological challenges.^9^ Timely referral and diagnosis are particularly important as early intervention leads to superior disease outcomes and potential reductions in radiographic progression.^10^ The current literature highlights the need to focus on solutions that will move us towards a recent UK community-defined (that is, patients, axial SpA clinicians, policymakers, social marketing experts, and health journalists) gold standard time to diagnosis of axial SpA of one year.^11^

## Challenges and experiences in primary care

A complex array of factors challenge the early identification and referral of axial SpA and contribute to diagnostic delay. The most pertinent challenges in primary care include: the fact that back pain due to axial SpA is a relatively uncommon cause of a common symptom; the existence of common misconceptions (for example, axial SpA is a predominantly male disease); poor healthcare professional (HCP) knowledge and awareness; and, to a lesser extent, the lack of disease-specific biomarkers.^12^

The journey to diagnosis can be frustrating, with patients trying to understand the fluctuating nature of their symptoms and feeling disbelieved and dismissed by HCPs.^13^ Patients describe having to ‘fight’ to be referred to a specialist and that they suffer psychologically (that is, desperation, depression, dejection, and distress) as a consequence of diagnostic delay.^13^ In a UK study, the complexities of an axial SpA diagnosis (for example, no pathognomonic features or the misattribution of back pain to mechanical MSK issues or fibromyalgia) are seen as a barrier to timely diagnosis.^14^ Suboptimal healthcare practice (for example, insufficient consultation time, lack of defined referral pathway, and poor communication between healthcare services), patient characteristics, and issues with patient/HCP interaction were also described as contributory factors.^14^ Patients often see a range of HCPs multiple times prior to diagnosis;^15^^,^^16^ survey data from two urban NHS axial SpA services have shown that some patients have visited a GP more than 10 times (1–5 visits, 63%; 5–10 visits, 23%; >10 visits, 14%) between symptom onset and diagnosis.^15^

For females with axial SpA, the patient journey may be longer and more demanding.^16^ Data from 13 European countries demonstrate that females attend a greater number of physiotherapist and osteopath appointments prior to diagnosis and are less frequently HLA-B27 positive compared with their male counterparts.^16^ The authors suggest that physician bias (that is, perceiving axial SpA as a male-only disease), underestimating symptoms, and a lower frequency of HLA-B27 (a core feature of the Assessment of SpondyloArthritis international Society [ASAS] classification criteria) may lead to inappropriate referrals to HCPs other than a rheumatologist.^16^ Females may experience greater disease burden (higher disease activity and poorer psychological health) compared with males.^16^ Thus, to reduce disease burden, decrease diagnostic delay, initiate timely disease management, and minimise unnecessary healthcare visits, it is crucial that HCPs recognise axial SpA symptomatology and the possible sex differences in axial SpA presentation.

Reducing diagnostic delay in axial SpA is challenging, particularly given the increasing clinical demand and pressures in primary care.^14^ To alleviate pressures on GPs and primary care, specialist physiotherapists in first-contact practitioner (FCP) roles are being deployed within general practice to assess individuals with MSK problems.^17^ FCPs have the potential to reduce diagnostic delay in axial SpA. However, gaps in the knowledge of axial SpA risk factors and features, along with a lack of awareness of national referral guidance, limit the clinical reasoning of FCPs.^17^ Amidst the chaos, PCPs need educational support and digital solutions to help recognise inflammatory back pain (IBP) and/or axial SpA efficiently, and facilitate quick and appropriate referrals to rheumatology.

## Educational resources to raise awareness and clinical suspicion of axial SpA: joining the dots

PCPs are experts at assessing individuals quickly and identifying key symptoms for efficient referral and management. However, axial SpA is challenging to diagnose and involves the recognition of a pattern of features, identified from patient history taking, physical examination, laboratory testing (for example, HLA-B27), and imaging modalities. There are no pathognomonic features of axial SpA, thus a hybrid approach to early identification is needed.

Initially, screening for IBP may be a useful approach for those presenting with persistent back pain. The ASAS IBP criteria include: an age of onset less than 40 years; insidious onset; improvement with exercise; no improvement with rest; and pain at night that improves upon getting out of bed.^18^ The IBP criteria are shown to have a sensitivity of 100%, a specificity of 60.1%, and a positive predictive value of 37.6% when ≥2 parameters are met in a primary care chronic low back pain population.^19^ However, not all individuals with axial SpA will present with IBP symptoms.

The National Institute for Health and Care Excellence (NICE) NG65 guideline can be utilised when suspecting axial SpA.^20^ It is essential to look for common features of axial SpA such as current or past pain at entheseal sites, peripheral MSK manifestations, and EMMs. Patient history could include symptom duration, alternating buttock pain, morning stiffness (>30 min), family history of SpA, response to non-steroidal anti-inflammatory drugs (NSAIDs), fatigue, physical activity, and smoking.

Laboratory and imaging tests may be supportive of suspicions, but it is important to note that inflammatory markers can be normal, HLA-B27 can be negative (especially in women), and sacroiliitis undetectable on plain film X-ray in people with axial SpA.^20^

Educational resources to support PCPs to identify axial SpA, and examples of service improvement projects can be seen in Box 1.

**Box 1.** Educational resources to support primary care practitioners

**Educational resource**	**Description**	**Weblink**
Act on Axial SpA — identification and referral in primary care	Quick presentations on axial SpA and IBP in primary care. Advice for GPs and FCPs. Links to additional resources	https://www.actonaxialspa.com/hcp-toolkit/
RCGP eLearning course — axial spondyloarthritis	A 60-minute educational course for GPs. Developed in partnership with NASS and BRITSpA	https://elearning.rcgp.org.uk/course/info.php?id=229
NASS Champions in Primary Care Programme	Service improvement projects and reflections A service improvement and leadership programme in association with the NHS Transformation Unit	https://www.actonaxialspa.com/champions-in-primary-care-projects-reflections/ https://www.actonaxialspa.com/champions-in-primary-care-programme/

*BRITSpA = British Society for SpondyloArthritis. FCP = first-contact practitioner. IBP = inflammatory back pain. NASS = National Axial Spondyloarthritis Society. RCGP = Royal College of General Practitioners. SpA = spondyloarthritis. SPADE = SPondyloArthritis Diagnosis Evaluation.*

## Digital solutions to aid identification, triage, assessment, and referral

The National Axial Spondyloarthritis Society (NASS) online symptom checker (https://www.actonaxialspa.com/symptoms-checker/) is freely available to the public as a screening tool for axial SpA. PCPs should be aware of the symptom checker and the output patients may bring to primary care consultations. It draws together the three validated sets of IBP criteria into a single set.^18^^,^^22^^,^^23^ A patient-facing poster can be printed for primary care waiting rooms to help drive individuals to the symptom checker and increase public awareness (https://www.actonaxialspa.com/hcp-toolkit/).

The recently updated Ardens MSK templates for suspected axial SpA and the PRIMIS pop-up alert tool can be used to support PCPs with their consultation data collection, clinical reasoning, and referral of suspected axial SpA in a real-time setting (Box 2).

**Box 2.** IT solutions to clinically support primary care practitioners

**IT solution**	**Description**	**Weblink**
Ardens Musculoskeletal Template	Updated axial SpA MSK templates to support PCPs in the assessment and triage of suspected axial SpA. Supported on EMIS Web and TPP’s SystemOne GP IT systems. Links to the NICE SpA guidance, ASAS criteria, SPADE tool, and the GIRFT axial SpA pathway	https://support-ew.ardens.org.uk/support/solutions/articles/31000163282-musculoskeletal-template https://www.actonaxialspa.com/ardens-templates-update/
PRIMIS pop-up tool	A pop-up alert tool to support the early referral of individuals with suspected axial SpA. Supported on EMIS Web and TPP’s SystemOne GP IT systems	https://www.nottingham.ac.uk/primis/projects.aspx
SPADE tool	A rapid evaluation tool completed by the PCP to help define the likelihood of axial SpA. A 12-point checklist with graphical output	http://www.spadetool.co.uk/

*ASAS = Assessment of SpondyloArthritis international Society. Axial SpA = axial spondyloarthritis. GIRFT = Getting It Right First Time. MSK = musculoskeletal. NICE = National Institute for Health and Care Excellence. PCP = primary care physician. SpA = spondyloarthritis. SPADE = SPondyloArthritis Diagnosis Evaluation.*

Within a busy clinic, PCPs can quickly complete the SPondyloArthritis Diagnosis Evaluation (SPADE) tool to help determine the likelihood of axial SpA in individuals with chronic back pain, without definitive changes on X-ray, and below the age of 45 years. The SPADE tool is a rapid, 12-point online checklist that provides a graphic on the probability of axial SpA and the recommendation of further tests (Box 2).

## Referral guidelines to support primary care practitioners

In the UK, a lack of a clear referral pathway is stated by HCPs as a barrier to diagnosing axial SpA.^14^ While there are many axial SpA referral strategies developed for use in primary care settings,^12^ local processes and pathways are likely to differ.

A printable infographic has been developed for PCPs that clearly summarises the NICE SpA guidance for identifying and referring spondyloarthritis.^21^ Using the infographic within clinic can remind clinicians of axial SpA signs and symptoms, and keep axial SpA at the forefront of clinical reasoning, especially for young adults presenting with persistent back pain (>3 months).

GPs are also encouraged to use NHS Advice and Guidance services to obtain specialist advice before or instead of referral. Using Advice and Guidance services may reduce inappropriate referrals (and/or investigations), facilitate learning, and improve interactions between PCPs and specialists. Study days and webinars may also promote discussion between primary and secondary care teams.

Further, PCPs can now refer to a new axial SpA pathway developed by NHS England’s Getting It Right First Time (GIRFT) rheumatology programme, NASS, and the NHS Best MSK Health Collaborative, which exemplifies best-practice processes and key clinician decisions. This pathway also proposes important information to include within an ‘advice and guidance’ request (Box 3).

**Box 3.** Referral guidelines to support primary care practitioners

**Referral tool**	**Description**	**Weblink**
NICE NG65 infographic	An infographic/poster summarising the NICE NG65 guidance^20^ for identifying and referring SpA	https://www.bmj.com/content/356/bmj.j839 ^21^
Advice and Guidance	NHS England Advice and Guidance services to provide the requesting clinician with specialist advice and reduce unnecessary hospital referrals. Links to information and toolkit	https://www.england.nhs.uk/elective-care-transformation/best-practice-solutions/advice-and-guidance/
GIRFT pathway	The GIRFT Rheumatology (in association with NASS and the Best MSK Health Collaborative) axial SpA pathway to support early assessment and quality of care	https://gettingitrightfirsttime.co.uk/wp-content/uploads/2022/11/Rheumatology-Axial-Spondyloarthritis-Pathway.pdf
GP referral template for chiropractors and osteopaths	An editable GP referral template to support chiropractors and osteopaths to recommend referral into rheumatology via a GP. Developed by NASS, the RCC, and the iO and endorsed by the RCGP and the CSP	https://www.actonaxialspa.com/hcp-toolkit/

*axial SpA = axial spondyloarthritis. CSP = Chartered Society of Physiotherapy. GIRFT = Getting It Right First Time. iO = Institute of Osteopathy. MSK = musculoskeletal. NASS = National Axial Spondyloarthritis Society. NICE = National Institute for Health and Care Excellence. RCC = Royal College of Chiropractors. RCGP = Royal College of General Practitioners. SpA = spondyloarthritis.*

Chiropractors and osteopaths report reluctance by GPs to accept their professional opinion and see this as a key perceived barrier to onward referral via a GP.^24^ GPs should be aware of and consider recommendations of referral into rheumatology from chiropractors and osteopaths. Such professionals can now use the editable GP referral template (Box 3).

## Conclusion

Delay to diagnosis in axial SpA remains a significant issue; PCPs can play a crucial role in reducing diagnostic delay. Early identification and referral of a person with suspected axial SpA can minimise psychological distress, morbidity associated with pain and stiffness, work instability, and maximise the opportunity to receive effective therapies earlier in the disease course. Educational resources are available for PCPs aiming to improve their knowledge of axial SpA. Digital solutions such as the Ardens MSK template, the PRIMIS pop-up tool, and the SPADE tool can be used to facilitate efficiency and clinical reasoning. The awareness of local referral pathways, as well as resources exemplifying best-practice processes (for example, the GIRFT axial SpA pathway) are key. To move us towards a UK gold standard time to diagnosis of 1 year, there is a need to reduce referral time from primary care to rheumatology. This requires improving the awareness of axial SpA within the primary care community, increased uptake of clinical tools, implementation of standardised rheumatology referral processes, and greater time allocation to optimise recognition and referral (for example, more staff, greater consultation time, and opportunities for education).
